# Blackberries and Mulberries: Berries with Significant Health-Promoting Properties

**DOI:** 10.3390/ijms241512024

**Published:** 2023-07-27

**Authors:** Mariana S. Martins, Ana C. Gonçalves, Gilberto Alves, Luís R. Silva

**Affiliations:** 1 CICS-UBI—Health Sciences Research Centre, University of Beira Interior, 6201-001 Covilhã, Portugal; mariana.sofia.morgado.martins@ubi.pt (M.S.M.); anacarolinagoncalves@sapo.pt (A.C.G.); gilberto@fcsaude.ubi.pt (G.A.); 2 CIBIT—Coimbra Institute for Biomedical Imaging and Translational Research, University of Coimbra, 3000-548 Coimbra, Portugal; 3 CPIRN-UDI/IPG—Center of Potential and Innovation of Natural Resources, Research Unit for Inland Development (UDI), Polytechnic Institute of Guarda, 6300-559 Guarda, Portugal; 4 Chemical Process Engineering and Forest Products Research Centre, Department of Chemical Engineering, Pólo II—Pinhal de Marrocos, University of Coimbra, 3030-790 Coimbra, Portugal

**Keywords:** blackberry, mulberry, phytochemicals, phenolic compounds, health-promoting properties

## Abstract

Blackberries and mulberries are small and perishable fruits that provide significant health benefits when consumed. In reality, both are rich in phytochemicals, such as phenolics and volatile compounds, and micronutrients, such as vitamins. All the compounds are well-known thanks to their medicinal and pharmacological properties, namely antioxidant, anti-inflammatory, anti-cancer, antiviral, and cardiovascular properties. Nevertheless, variables such as genotype, production conditions, fruit ripening stage, harvesting time, post-harvest storage, and climate conditions influence their nutritional composition and economic value. Given these facts, the current review focuses on the nutritional and chemical composition, as well as the health benefits, of two blackberry species (*Rubus fruticosus* L., and *Rubus ulmifolius* Schott) and one mulberry species (*Morus nigra* L.).

## 1. Introduction

Fruit consumption is promoted globally, being considered an essential part of any diet because it helps people to ingest more vitamins, minerals, dietary fiber, and phytochemicals. Therefore, it should not be surprising that recent epidemiological and clinical studies have shown the importance of a fruit-rich diet for the prevention of many illnesses, including cardiovascular diseases, cancer, and metabolic disorders [1,2]. The World Health Organization (WHO) suggests that individuals have a minimum intake of 400 g of fruits (five servings) per day in order to prevent chronic diseases and other illnesses, as well as to prevent micronutrient deficiencies [1,3].

All fruits have gained in appeal and interest over the past few decades. Among them, red fruits from many families, such as Rosaceae (strawberry, raspberry, blackberry, and sweet cherry), Ericaceae (blueberry, cranberry), and *Moraceae* (*mulberry*) have received special attention, due to their high nutritive value, distinctive taste, flavor, and nutraceutical properties, as well as their health-promoting properties [4].

**Figure 1:**
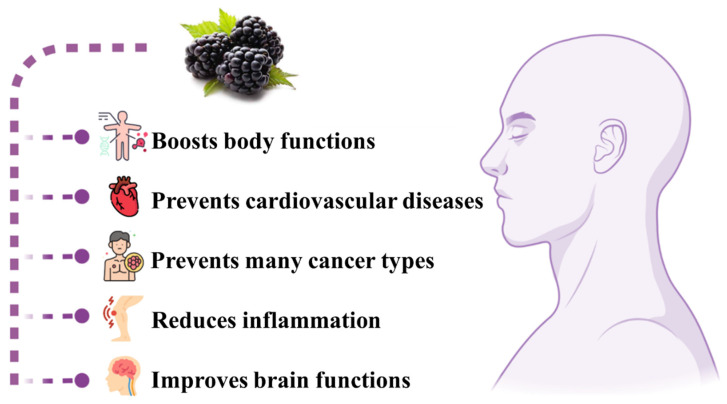
The main benefits linked to blackberries and mulberries consumption.

Vitamins A, C, and E, minerals (calcium, phosphorus, iron, magnesium, potassium, sodium, manganese, and copper), dietary fiber, and phenolics are just a few of the bioactive compounds and nutrients found in these fruits. Among them, this last subclass has undergone extensive research mainly due to its notable anti-inflammatory and antioxidant properties [5,6]. Indeed, phenolics are considered the main factors responsible for the health benefits attributed to these berries, and stand out due to their capacity to prevent cardiovascular diseases [7], reduce inflammation [8], improve neurological function and boost immune system [9], and offer resistance against oxidative stress (Figure 1) [10].

As well as those of other fruits and vegetables, the contents of red fruits also differ in terms of their nutritional value, consumer acceptability, and qualitative and quantitative composition depending on the species, cultivar, genotype, maturity stage, agricultural practices, environmental factors, soil conditions, and subsequent storage conditions [4,11].

In light of these facts, as well as the rising economic value of red fruits, the current review focuses on the nutritional and chemical composition, as well as the health benefits, of two blackberry species (*Rubus fruticosus* L.) and (*Rubus ulmifolius* Schott) and one mulberry species (*Morus nigra* L.).

## 2. *Rubus fruticosus*, *Rubus ulmifolius* and *Morus nigra*

**Figure 2:**
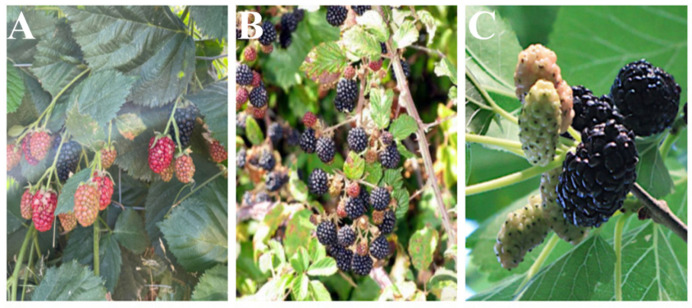
(**A**) *Rubus fruticosus*, (**B**) *Rubus ulmifolius* [14], (**C**) *Morus nigra* [15].

Focusing on *R. fruticosus* L. and *R. ulmifolius*, both are semi-prostrate erect, scrambling, and perennial deciduous prickly fruits whose shrubs grow up to 3 m at a rapid rate [12]. Their stems are up to 7 m long and are stretched out nearly upright with leaves [6]. Unlike *R. ulmifolius*, *Rubus fruticosus* is a cultivated shrub with no thorns. In addition, *R. ulmifolius* is widespread in forests, hedges, and deserted fields, and along water lines, walls, and fences, and its stems are thorny (Figure 2A,B) [13].

Blackberries are the red fruits of this shrub. This species, a drupe-like aggregate fruit composed of numerous drupelets, belongs to the Rosaceae family, subfamily *Rosoideae* and genus *Rubus*, and has a morphology similar to that of raspberries. *Rubus* has over 740 species and 12 subgenera worldwide [6,7,16].

There are presently around 40 distinct species of blackberries worldwide, but regions with mild winters and long temperate summers are better suited for their development [17,18]. It is believed that this plant originates from Armenia. On a worldwide basis, blackberries are becoming increasingly popular, and are mostly farmed in North America, Europe, Asia, South America, Central America, and Africa [14,17]. The main blackberry-growing regions in Europe are Serbia and Hungary, with Serbia accounting for 90% of processed and exported production [19]. Furthermore, the yield of wild blackberries is significant, accounting for 154,000 tonnes in 2005 [20]. The United States of America is the world’s top producer of blackberries, with a production that, in 2017, reached a value of USD 31 million [21]. In 2020, Portugal exported around 29,848 tonnes of raspberries, strawberries, and mulberries [22]. In fact, the world production of these small fruits is growing due to the new trend towards biological products and the growing interest in their nutritional characteristics. They are rich in antioxidants and fiber, vitamins A, B, C, E, and K, calcium, magnesium, and potassium, and are beneficial to promoting health status at many levels [23,24,25].

Commonly, blackberry fruits have been typically consumed fresh or frozen/processed, including when made as jams, juices, syrups, and wines [24,25].

Another factor that influences phenolic concentrations is the food processing method used to produce a product that customers will want to buy. The antioxidant potential significantly decreases as a consequence of jam manufacturing. The principal cause of these declines is the inclusion of glucose-fructose syrup. Total phenolic compounds, total flavonoids and monomeric anthocyanins, and total antioxidant capacity values were found to be lower (between 76–89%) after the addition of glucose-fructose syrup than those recorded in the frozen sample [26].

In addition to its versatility, blackberry fruit is particularly valuable to producers due to its low cost of production and cultivation [27]. Since the fruit’s external appearance and internal quality are directly linked to the amount of primary and secondary metabolites present, fruit quality is crucial to both consumers and the food industry. It is also essential to remember that fruit with higher quality has a higher market value. Smaller fruits are firmer because they have the same number of cells as larger fruits, giving a higher density to the plant tissue. Fruit size is typically negatively correlated with firmness and berry phenolic content [18,19].

This fruit has the highest quality and flavor when it is fully ripe. From a business perspective, the color of the fruit and juice is crucial, because customers evaluate products based on their visual appearance. The color of blackberry fruits is influenced by a number of variables, including genotype, production conditions, fruit ripening stage, harvesting time, climate, soil, and storage conditions [28,29]. In terms of climate, some environmental elements influence the fruit composition, which is defined by the presence of substances known as nutraceuticals, which offer health advantages and assist in the treatment of disorders [30].

Blackberries and their by-products have been used since ancient times in traditional medicine, but recently the knowledge concerning their health-promoting components has received a lot of attention, particularly due to their richness in different bioactive compounds, with the presence of vitamins, minerals, fiber, and phenolic compounds standing out [19,23,25,31]. Therefore, it is not surprising that consumers favor the nutritional and antioxidant qualities associated with these fruits [4]. These characteristics depend on the region, variety, and time of harvest [32,33]. Additionally, blackberry phenolic content can also be affected by soil composition, which, in turn, results in variations between cultivars produced in the same area [5,18].

The fruit of M. nigra, a member of the *Moraceae* family and the genus Morus, is frequently compared with that of *R. fruticosus* and *R. ulmifolius* (Figure 2A–C) [25,26]. Although these three species have comparable appearances and chemical properties, and are consumed fresh, as well as processed to make jam, marmalade, syrup, a variety of soft beverages, and traditional items, *M. nigra* develops from trees that can reach a height of 10–13 m and exhibits higher potential for adaptation to diverse soil and environmental conditions [21,22]. Their origin was India and China, but nowadays, they are commonly found in Asia, Europe, America, and Africa [21,24]. They have a wide range of varieties; however, the three most popular types are black mulberry (M. nigra), white mulberry (*M. alba*), and red mulberry (M. rubra) [33]. Among these, the black mulberry is an edible fruit that is 2–3 cm long, with a complex cluster of several tiny drupes, and is dark purple, almost black, when completely mature. In Xinjiang, a region of China, and Eastern Anatolia, a region of Turkey, black mulberry fruits are used as a traditional medicine for the prevention and treatment of hypertension, tonsillitis, sore throat, anemia, and iron deficiency [34,35]. According to recent studies, black mulberries have more flavonoids, anthocyanins, and antioxidant abilities than red or white mulberries [26,32]. Since this fruit has a high concentration of naturally occurring phenolic compounds, such as phenolic acids, flavonols, and anthocyanins, it shows a wide range of biochemical activities, including antioxidant, anti-hyperlipidemia, and anticancer properties [33,36,37].

## 3. Nutritional and Chemical Composition

Berries, including blackberries and mulberries have high nutritional content, including of fatty and organic acids, minerals (Mg, Fe, K, and Ca), vitamins (A, B, C, K, and E), proteins and amino acids, and carbohydrates (sugars, and fiber) [16,24,27,32]. In addition, and focusing on blackberries and mulberries, they are also a great source of bioactive compounds with pharmaceutical potential, including phenolics (e.g., anthocyanins, hydroxycinnamic acids, and flavonols) and volatiles [13,25,30,33,35,36]. As already mentioned, and like other fruits, the nutritional content and quality of blackberries and mulberries are influenced by their chemical composition [37]. Additionally, the physiological age of red fruits at harvest has a significant impact on post-harvest quality, having noticeable variations in colour, hardness, acidity, and TSS as both berries grow [24,30,37,38]. Several studies identified several characteristics of farmed and wild blackberry fruits [17,38]. In general, the majority of quality assessments are based on the sugar/acid ratio level, calculated from total soluble solid (TSS; °Brix) and titratable acidity (TA). The TSS parameter indicates sugar content in fruits, while pH and TA represent total acids that contribute to sweetness and acidity, respectively, of fruits and related products [39,40].

Berry weight ranges from 1.2 g to 5.4 g for cultivated blackberries, such as R. fruticosus, whereas in wild blackberries, e.g., R. ulmifolius, it varies from 0.4 g to 1.2 g. This indicates that cultivated berries have a higher mean weight compared to wild genotypes. A similar trend is observed for length and width. However, TSS values are lower in cultivated fruits (8.6%–14.1%) than in wild genotypes (12.9%–22.3%). The mean TSS of wild genotypes is around 20%, whereas the mean pH of the wild genotypes is higher than that of the cultivated genotypes [38]. Concerning ash content, *R. ulmifolius* possesses around 0.58 g per 100 g fresh weight (fw). A higher degree of moisture was found for wild blackberries, with a value of 70 g per 100 g fw [13].

The weight of *M. nigra* ranges from 4.18 g to 5.55 g, while moisture content is around 78.03 fw and is the highest found on the *Morus* species [30,35,41]. Total ash content is around 0.50 g per 100 g dry weight (dw), whereas pH values range from 3.43 to 4.78 [35,42] and TA between 0.17% and 1.97% [43,44]. Recently, it was reported, in Chinese mulberries, TA values were between 5.82 and 48.49 mg citric acid per g fw [37]. The TSS content fluctuates between 6.20 to 19.43 °Brix [37,44].

### 3.1. Macronutrients

**Figure 3:**
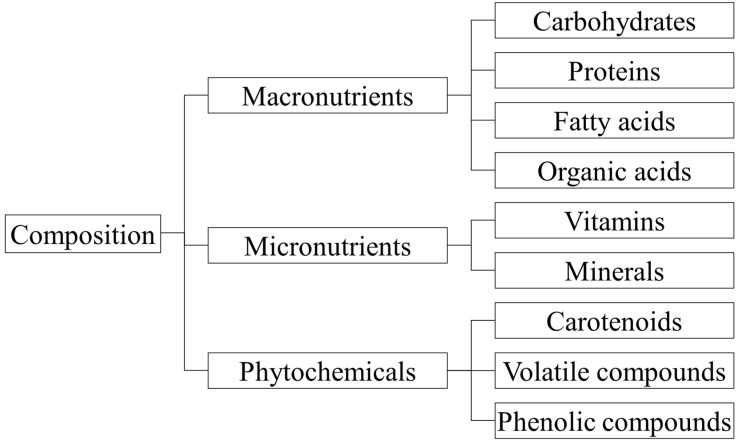
General composition of fruits.

Macronutrients are chemicals that humans ingest in large quantities and are the primary body source of energy. The most well-known are carbohydrates, proteins, and organic and fatty acids (Figure 3). Among these, carbohydrates are considered the main source of energy used by human organisms. However, all of them are considered vital for preserving our health and life [45,46].

#### 3.1.1. Carbohydrates

Sugars are essential to a fruit’s general taste character, nutritional value, and caloric density. They are the primary result of photosynthesis, and are required for the development of plant cell walls, energy production, and the formation of a number of signaling molecules at cellular and tissue levels, participating in the formation of aroma compounds [41]. Since most customers prefer sweet fruits, a higher fructose concentration is preferred because fructose is typically sweeter than glucose and sucrose [24].

Fructose, glucose, sucrose, trehalose, and raffinose are found in *R. fruticosus* and *M. nigra*. Comparing both, *M. nigra* contains more total and reduced sugars, but lower levels of saccharose (Table 1) [25,41,47,48,49].

The carbohydrates most found in blackberries and mulberries are glucose and fructose. Among these, fructose is the most abundant [47,48].

#### 3.1.2. Proteins and Amino Acids

Proteins are chains of amino acids linked together by peptide linkages. Proteins are essential in the human organism. They can heal cells and structures, providing structural support, and contribute to pH and fluid equilibrium. They also enhance the immune system by transporting and storing nutrients and providing energy when needed [45]. Although fruits are not considered an excellent source of proteins, these berries present considerable amounts of proteins when compared to other fruits, with amounts around 1.39–2.4 proteins per 100 g for blackberries and about 1.44 g per 100 g for mulberries (Table 1) [47,48].

#### 3.1.3. Fiber

Fiber is classified into (i) water-soluble fiber and (ii) insoluble fiber. Soluble fiber delays digestion and improves nutrient uptake. By restricting the enterohepatic circulation of cholesterol, soluble and insoluble fibers improve gut health and reduce the risk of cardiovascular diseases [45].

Dietary fiber is a non-caloric carbohydrate that human small intestines cannot process or ingest. Fruits contain dietary fiber, particularly soluble fiber, in quantities higher than 7%, and, therefore, they can reduce the risk of cardiovascular and coronary heart diseases. Thus, the primary nutritional reason for including fruits in a healthy diet is due to their fiber content, principally due to their gastrointestinal regulatory abilities, which contribute to human health maintenance. Additionally, fiber works together with vitamins, increasing the biological activities of foods [45].

Among berries, blackberries present the higher fiber content (approximately 5.3 g per 100 g) (Table 1). On the other hand, black mulberries only possess around 1.7 g per 100 g [47,48].

#### 3.1.4. Fatty Acids

Fatty acids are part of triglycerides, and are the principal form in which fat occurs. Fatty acids can exist naturally, presenting different chain lengths and double bonds. They may be saturated, monounsaturated, or polyunsaturated. Fatty acids are required for the formation and reparation of cell structures, including cell walls. In addition, they are crucial to human well-being [45].

Blackberries have extremely little fatty acid content, with saturated fats making up about 0.014 g per 100 g, monounsaturated fats around 0.047 g per 100 g, and polyunsaturated fats approximately 0.28 g per 100 g of fruit (Table 1) [47,48]. Concerning M. nigra, they contain oleic acid (26.0%), palmitic acid (23.8%), and linoleic acid (23.1%) [34]. However, their percentages are widely variable. For example, Jiang and Nile [30] reported that the average linoleic acid concentration is 4.1 times higher than that of palmitic acid and 4.8 times higher than that of oleic acid of *M. nigra* from Xinjiang, a province of China. These variations could be attributed to different cultivars, as well as the ecological circumstances under which the species are produced [30,34,35].

#### 3.1.5. Organic Acids

Organic acids are primary metabolites found in abundance in all plants, particularly in fruits and vegetables. The most well-known include citric, malic, and galacturonic acids. These compounds have a significant impact on the organoleptic properties of fruits and vegetables, particularly flavour, colour, and aroma [13,36]. When the fruit is immature, it has a greater acid content, which decreases with the harvest. Organic acids are available in free form and help to stabilise anthocyanins [50].

These primary metabolites can also inhibit the development of microorganisms in fruit juices, thereby improving product quality preservation [45].

The total quantity of organic acids found in several species of berries has been reported to range from 21.5 to 235 mmol/kg. The *R. fruticosus* species is the one that presents the highest content (45.1 mmol/kg) [51].

*Rubus ulmifolius* presents oxalic, quinic, malic, shikimic, ascorbic, and fumaric acids (Table 2), accounting for around 238 mg per 100 g fw. Quinic acid is the compound with the highest concentration (119 mg per 100 g fw), followed by oxalic (71 mg per 100 g fw), malic (29 mg per 100 g fw), shikimic (11.33 mg per 100 g fw), and ascorbic acids (6.66 mg per 100 g fw); fumaric acid is only detected in trace amounts [13]. On the other hand, the organic acids found in *R. fruticosus* are citric, oxalic, malic, ascorbic, and fumaric acids. Malic acid is predominant (5706.37 mg per 100 g dw), while ascorbic acid is the lowest (6.00 mg per 100 g dw). Other organic acids, namely, quinic, shikimic, tartaric, and succinic acids, have not been identified [52].

Relative to black mulberry fruits, these contain a variety of organic acids (Table 2). To date, citric, tartaric, malic, and succinic acids are the only organic acids detected in *M. alba*, *M. nigra*, and *M. rubra* species [36].

### 3.2. Micronutrients

Although micronutrients (e.g., vitamins and minerals) are consumed in small amounts, they are essential for health and vital functions [45]. They are essential elements that the organism requires to stay healthy (Figure 3). This requirement is determined by each person’s unique needs, varying according to various metabolic circumstances throughout the life cycle (age, lifestyle, hormonal activity, exercise, etc.) [53]. All of the essential micronutrients cannot be synthesized within the body, and are supplied by the diet. As a result, a diverse range of foods is important in our nutrition [48].

#### 3.2.1. Minerals

A sufficient mineral intake is needed for good nutrition and food quality, and to avoid chronic nutrition-related illnesses. Certain elements, such as calcium (Ca), iron (Fe), and zinc (Zn), are deficient in certain populations [48]. Fruit mineral composition is affected by growth circumstances, such as soil and geographical location, as well as species or varieties [41].

A total of ten minerals have been reported in raw blackberries, namely, Ca, Fe, magnesium (Mg), phosphorus (P), potassium (K), sodium (Na), Zn, copper (Cu), manganese (Mn), and selenium (Se) [47,48]. Black mulberry possesses all the minerals mentioned above (Table 1), with K, P and Ca found in higher concentrations [30,34,42,47,48]. In particular, Ca is necessary for the growth of bones and muscles, while Fe is required for the formation of hemoglobin, and to help oxygen and electron transfer [45,54].

#### 3.2.2. Vitamins

Vitamins are complex organic essential compounds that are classified into two types: (i) fat-soluble and (ii) water-soluble [55]. They are required for the organism’s functions and normal growth. Each vitamin serves a particular purpose in regular metabolism, development, vitality processes, and energy transformation. Furthermore, some of them are antioxidants. Fruits are without a doubt the most significant source of vitamins in the human diet [45].

In particular, blackberries have higher levels of vitamins C and K. Vitamin C is a water-soluble vitamin that is present in higher amounts in fruits and vegetables, which contain up to 50% [54]. It is known that blackberries contain around 21 mg per 100 g of vitamin C, whereas black mulberries contain 17.41–28.33 mg per 100 g of fruit [30,33,41,43,56,57]. The amount of vitamin K in blackberries is approximately 19.79 mg per 100 g (Table 3) [47]. This vitamin can help the human body to fight against free radicals. Furthermore, blackberries contain approximately 1.17 mg of vitamin E per 100 g (Table 1) [47]. This vitamin can serve as a safeguard and protect the human body from free radicals, as well as strengthening the immune system and retarding skin aging. Finally, diets with higher amounts of vitamin C may reduce the risk of acquiring various types of malignancies, e.g., cardiovascular diseases and sicknesses caused by environmental factors [54,55].

#### 3.2.3. Tocopherols

Vitamin E consists of the generic denomination of eight liposoluble compounds, alpha (*α*), beta (*β*), gamma (*γ*) and delta (*δ*)-tocopherols, each of which has specific biological activities. Among these, *α*-tocopherol is the compound with the highest antioxidant capacity [13]. The function of vitamin E as an antioxidant in the peroxidation of cell membranes occurs by supplying a hydrogen atom to the peroxide radical formed, acting as a scavenger of free radicals, hence protecting cell membranes from possible damage. Vitamin E is mainly found in products rich in fat, such as almonds, vegetable oils, and some fruits and vegetables. Blackberry exhibits very small amounts of tocopherols which can be explained by the low amounts of fat found in this fruit [58]. Blackberry fruit contains all of the tocopherols’ isoforms, with *γ*-and *δ*-tocopherol being present at higher concentrations.

Tocopherols found in *R. ulmifolius* are described in Table 4. Isoforms, namely, *α*-, *β*-, *γ*-, and *δ*-tocopherol are present, representing quantities of 5.1–13.48 mg per 100 g fw [13]. *γ*-Tocopherol was highlighted as a major isoform present, with a concentration ranging from 1.34 to 3.73 mg per 100 g fw, followed by *δ*-tocopherol and *α*-tocopherol with similar contents (0.9–3.69 and 1.15–3.38 mg per 100 g fw, respectively) [13,58]. *β*-Tocopherol is detected at low concentrations (values of 0.020–0.24 mg per 100 g fw) [13].

In *R. fruticosus*, only *α*-tocopherol was found, in a concentration of 610 mg per 100 g. On the other hand, *M. nigra* shows nearly seven times more *α*-tocopherol than *R. fruticosus*. Additionally, in *M. nigra*, the four isoforms were found (Table 4), with the prevalence of *α*-tocopherol (4300 mg per 100 g), followed by *γ*-tocopherol (1250 mg per 100 g). *δ*-Tocopherol (550 mg per 100 g) and *β*-tocopherol (127 mg per 100 g) were less abundant [49,58].

In a general way, *γ*-tocopherol has been shown to be a highly effective molecule in postponing arterial thrombus development, lowering LDL oxidation and superoxide production, and avoiding lipid peroxidation. It has also been mentioned that regular consumption of food rich in this isoform reduces the risk of myocardial infarction and death from ischemic heart disease. Regarding antioxidant and protective effects of tocopherols, many studies focus primarily on *α*-tocopherol, which is the main form of vitamin E, in over-the-counter supplements [13,58].

### 3.3. Phytochemicals

**Figure 4:**
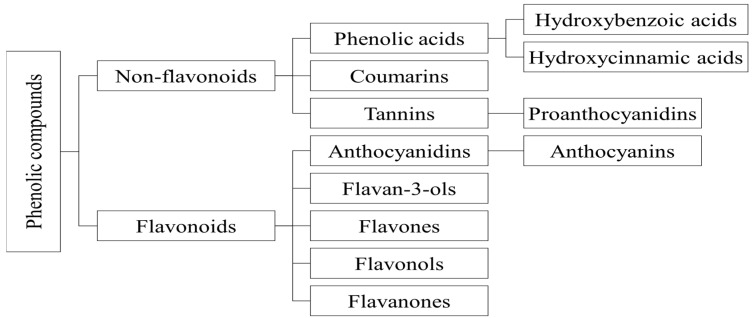
Phenolic compounds’ classification.

Phytochemicals are non-nutrient bioactive plant molecules found in fruits, vegetables, whole grains, and other plant foods [45,54]. Blackberries have a high amount of environmental variation due to their extensive geographic distribution, which influences their physical and chemical characteristics, and, hence, the profiles of bioactive substances, including anthocyanins, flavonoids, and carotenoids (Figure 4) [4,11,38]. *Rubus berries* are thought to be an abundant source of phytochemicals that play an important role in the prevention of modern chronic illnesses [19,36]. The physicochemical characteristics of mulberry cultivars are essential for economic and dietary benefits [19,20,21].

Phytochemicals are important antioxidants, having a positive impact on human health, particularly in the prevention of cardiovascular, inflammatory, and cancer diseases. Therefore, it is essential to identify and quantify the bioactive constituents of plant extracts because they are mainly responsible for the biological and pharmacological actions exhibited by foods [26,32,45].

#### 3.3.1. Carotenoids

Carotenoids are a class of fat-soluble natural pigments that have a variety of health benefits. These natural pigments metabolized by plants are responsible, along with anthocyanins, for the yellow, orange, and red colours in fruits and vegetables. The term carotenoid refers to a family of structurally similar pigments found primarily in plants [59]. Based on their functional groups, carotenoids are classified into two groups: (i) xanthophylls, which contain oxygen as a functional group (e.g., lutein and zeaxanthin), and (ii) carotenes, which contain only the parent hydrocarbon chain and no functional group, such as *α*-carotene, *β*-carotene, and lycopene [45].

Their content and types in plants are affected by several pre- and post-harvesting variables, genotype, ripening time, cultivation technique, climatic conditions, and processing methods [59]. Additionally, different parts of the same plant may also contain varying types and quantities of carotenoids. For example, the peel of fruits is typically higher in carotenoids than the pulp. Climate and growth circumstances can also have an impact on the quantity of carotenoids in plants. According to these findings, fruits exposed to higher temperatures and more sunlight may boost carotenoid production in order to defend the plant from photo-oxidation [45].

The daily ingestion of carotenoids is important to increase antioxidant activity, intercellular communication, gene regulation, and immune system activity. Indeed, carotenoid-rich diets have been linked to a lower incidence of many types of cancer, cardiovascular diseases, age-related macular degeneration, and cataract formation [45,48].

Unfortunately, when compared to other red fruits, such as blueberries and raspberries, the quantity of carotenoids in blackberries is small: 128 µg per 100 g of fruit (*β*-carotene) (Table 5) [13,47,60].

#### 3.3.2. Volatile Compounds

Flavour and aroma are two of the most essential aspects of fruits’ excellence and acceptance. The aroma of some fruits has been linked to their concentration of volatile organic compounds. They derive from fatty acids, amino acids, carotenoids, and phenolics [45]. Additionally, the metabolism of fruits produces volatile compounds during the ripening, harvesting, post-harvesting, and storage. As a result, the volatile composition of blackberries is affected by the genotype, origin, technological treatment (freezing, drying, among others), ripening stage, harvest, and storage conditions [61,62,63,64]. Therefore, the analysis of volatile compounds is critical for understanding the components responsible for their flavour and aroma, as well as the best harvest period for higher quality and phytosanitary qualities [4,61].

Although several volatile compounds exist, regarding blackberry fruits, aldehydes, alcohols, ketones, esters, hydrocarbons, terpenoids, furanones, and sulfur compounds are the main contributors to their aroma [8,65]. Hence, terpenoids (75.38%) are the most abundant chemical category of volatile chemicals in *R. fruticosus*, whereas aldehydes (0.53%) are the least abundant [65].

On the other hand, *R. ulmifolius* possesses around 33 different volatile compounds: nine aliphatic alcohols, three branched alcohols, six aldehydes, two ketones, six terpenoid compounds (including *β*-myricene, D-limonene, *β*-linalool, L-*α*-terpineol, sulcatol, and sulcatone), four compounds containing a benzene-ring (including methoxyphenyl oxime, methyl salicylate, benzyl alcohol, and phenylethyl alcohol), and ethyl octanoate (an ester), 2-methylbutanoic acid (a carboxylic acid), and 2-ethylfuran (a cyclic ether). This species of blackberry contains high amounts of benzenoids, aldehydes, and alcohols (Table 6) [62,66].

Focusing on M. nigra, a previous study determined the presence of 67 volatiles: five acids, twenty-five alcohols, two aldehydes, twenty-six esters, five hydrocarbons, one ketone, and three phenols. The most prevalent chemicals in samples were aliphatic alcohols, which accounted for 47.5% of the total volatile component. The majority of the alcohol was ethanol (82.3%). Furthermore, ten aliphatic alcohols (ethanol, 1-propanol, 2-butanol, 2,3-butanediol, 2-methyl-1-propanol, 2-methyl-1-butanol, 3-methyl-1-butanol, benzyl alcohol, phenylethyl alcohol, and terpene-4-ol) were also found. Surprisingly, although aldehydes are abundant in many fruits, only two aldehydes (acetaldehyde and benzaldehyde), accounting for only 2.1% of the total volatile compounds, were detected. Relative to *M. alba*, esters are largely found, representing 36.3% of all volatile compounds found in this mulberry. In addition, isovaleric acid (94.4%) was revealed to be the most abundant fatty acid [64].

Finally, using solid-phase microextraction and gas chromatography-mass spectrometry, 45 volatile compounds have been reported in R. fruticosus. Terpenoids made up the vast majority (97.7%), with limonene being the most frequent compound. The discovered volatiles extracted with hexane were largely hydrocarbons, whereas those extracted with acetone were furans and pyrans. Hexane-extracted volatiles were also identified, with the majority of the compounds being the aliphatic ones, and just 13% were aromatic. The identified compounds accounted for 82% of the overall peak area in the acetone extract chromatogram. Altogether, the most essential volatile components responsible for the blackberry flavor are heptanol and p-cymen-8-ol [65].

#### 3.3.3. Phenolic Compounds

Phenolic compounds can be classified into (i) non-flavonoids and (ii) flavonoids. Phenolic acids, coumarins, and tannins are examples of non-flavonoids. Flavonoids are further classified into five main subgroups: (i) anthocyanidins and their glycosides anthocyanins, (ii) flavan-3-ols, (iii) flavones, (iv) flavonols, and (v) flavanones (Figure 4). They are regarded as non-nutrient physiologically active molecules capable of functioning as free radical scavengers [45].

This subclass is composed of secondary metabolic products found in fruits, vegetables, leaves, nuts, seeds, flowers, and barks which are kept in cell structures of the fruit skin, pulp, and seeds of fruits [67]. They are essential for plant reproduction, development, and metabolism, as well as for defence against pathogenic viruses and infections [11,12]. In addition to their activities in plants, in our diet, phenolics may lower the risk of chronic illnesses, such as cancer, heart disease and diabetes [31,36,45,67]. As mentioned above, their content in berries may be influenced by genotype, geographic region, storage conditions, ripeness, and climate, among others [11,33,39,41,43]. According to a previous study, polyphenols steadily rise throughout the last phase of maturity in blackberry and mulberry fruits [34].

Table 7 lists the concentrated phenolic compounds from the three blackberry species reported in the literature.

##### Phenolic Acids

Phenolic acids are frequent and widespread bioactive molecules in nature. They are commonly found in bound forms, such as amides, esters, or glycosides, with the exception of caffeic and ferulic acids, which are mainly sterified with other molecules such as carbohydrates and organic acids [6].

There are two major groups of phenolic acids: hydroxybenzoic acid derivatives and hydroxycinnamic acid derivatives [45].

Hydroxycinnamic acids are composed of a nine-carbon structure (C6-C3) with a side-chain double bond (with *cis* or *trans* configuration). The most prevalent hydroxycinnamic acids are caffeic, *o*-coumaric, *p*-coumaric, *m*-coumaric, and ferulic acids [73,74].

**Figure 5:**
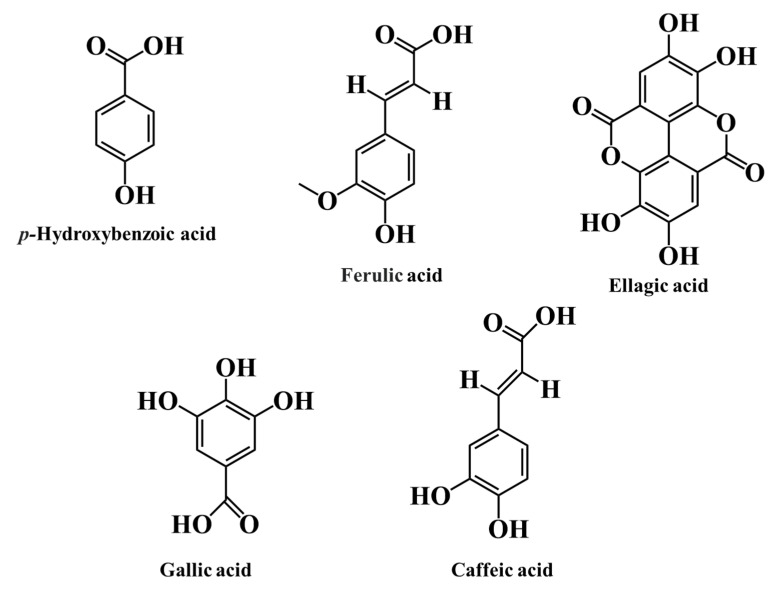
Principal phenolic acids found in *Rubus fruticosus*, *Rubus ulmifolius*, and *Morus nigra*.

In a general way, caffeic, ferulic, chlorogenic and *p*-coumaric acids were the main ones identified and quantified in both berries (Figure 5) [37,44,69,70]. Ferulic acid is predominately found in *R. ulmifolius* (388.59 µg per 100 g dw) (Table 7) [69]. Comparative to other red fruits, strawberries present higher amounts of *p*-coumaric acid (concentrations around 0.7–4.1 mg per 100 g fw, double that reported in R. fruticosus) [75].

Hydroxybenzoic acid is generated from cinnamic acid and is commonly found in food as esters with quinic acid or glucose. This subgroup of phenolic acids is produced from benzoic acid and has a typical common structure of C6-C1. *p*-Hydroxybenzoic, protocatechuic, vanillic, syringic, tannic, and gallic acids are the principal ones reported [45]. They form components of complex structures, such as lignins and hydrolysable tannins, and contribute to formation of cell walls and proteins [76]. In comparison to hydroxycinnamic acids, hydroxybenzoic acids are present in relatively modest concentrations in red fruits. Gallic acid is present in M. nigra, R. fruticosus, and *R. ulmifolius* in higher concentrations (21.83 to 40.90 mg per 100 g fw, 145.85 mg per 100 g fw, and 268.72 mg per 100 g fw, respectively) [44,65,68]. Comparing the three species, *R. ulmifolius* showed the largest level of this hydroxybenzoic acid. Relative to other red fruits, sweet cherries possess amounts fluctuating from 0.73 to 10.64 mg per 100 g of fw, and this concentration is much lower than that of blackberries and mulberries [77].

Concerning M. nigra, the major hydroxybenzoic acid present in this fruit is gallic acid (21.83 to 40.90 mg per 100 g fw), followed by ellagic acid (1.36 to 6.32 mg per 100 g fw) [44].

Although the precise role of phenolic acids is uncertain, it is known that they help with food intake, structural support, enzyme activity, protein synthesis, photosynthesis, and allelopathy. Phenolic acids are also the ancestor of bioactive compounds used in food, cosmetics, and pharmaceutical industries [74]. According to research, in individuals, this subclass of fruit compounds has the potential to improve brain function, protect against heart disease, and stop the growth of some cancers [73,74,78].

##### Flavonoids

Flavonoids are a subgroup of phenolic compounds that fall into several groups, such as anthocyanidins, flavan-3-ols, flavones, flavonols, and flavanones (Figure 4) [45].

**Figure 6:**
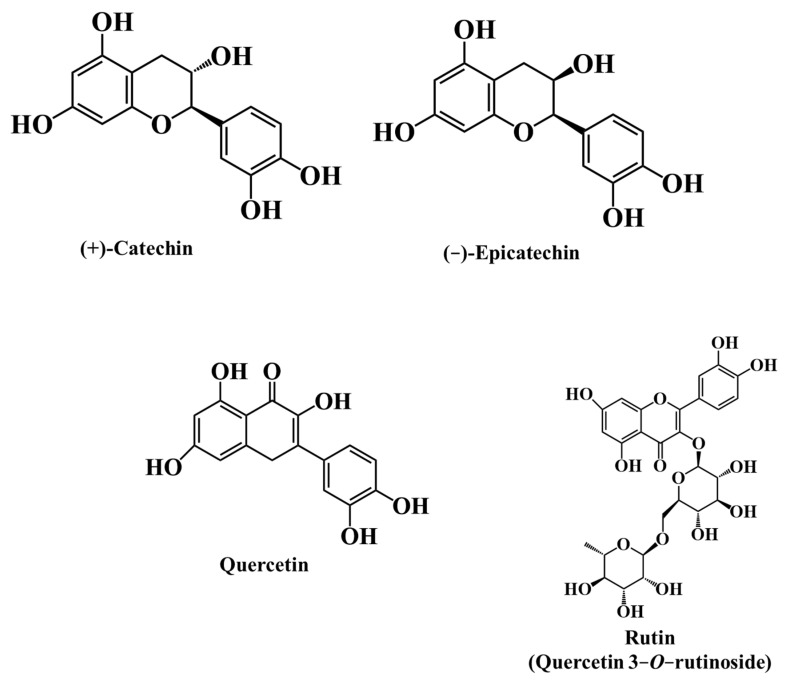
Principal flavan-3-ols and flavonols present in *Rubus fruticosus*, *Rubus ulmifolius*, and *Morus nigra*.

The total flavonoid content in *M. nigra* fruit is around 254.0 mg catechin equivalent per 100 g fw [79]. The predominant flavonoids reported in black mulberry fruits are rutin, quercetin, and (+)-catechin (Figure 6), with values varying from 32.06 to 133.60 mg per 100 g fw for rutin, followed by quercetin (2.33 to 11.25 mg per 100 g fw) and catechin (2.28 to 10.54 mg per 100 g fw) (Table 7) [29,41].

The total flavonoids of *R. fruticosus* fruit fluctuating from 30.4 to 82.2 mg catechin equivalent per 100 g of fw, with quercetin, rutin, (+)-catechin, (−)-epicatechin, and myricetin the most abundant [65,68,69,80]. In particular, the level of quercetin in blackberries (20.62 mg per 100 g) is significantly higher than that in black mulberries (2.33 to 11.25 mg per 100 g) (Table 7) [44,65].

Other flavonoids found in both berries are anthocyanins. These are considered the primary factor responsible for the color of many fruits and vegetables. Anthocyanins can be found in the cell at locations known as anthocyanoplasts, which are vacuole sites [81], and are responsible for the red, purple, and black pigments of fruits and vegetables, as well as being recognized for their notable health benefits [45]. The colors produced by anthocyanins depend on pH, light, and temperature, appearing reddish in more acidic conditions and turning blue as the pH rises [82].

The blackberry is an excellent source of natural antioxidants. Indeed, the total anthocyanin content in *R. fruticosus* ranges from 70 to 180 mg per g fw [83,84], while in R. ulmifolius, the total anthocyanin content ranges from 5.87 to 35.55 mg per g fw [85].

**Figure 7:**
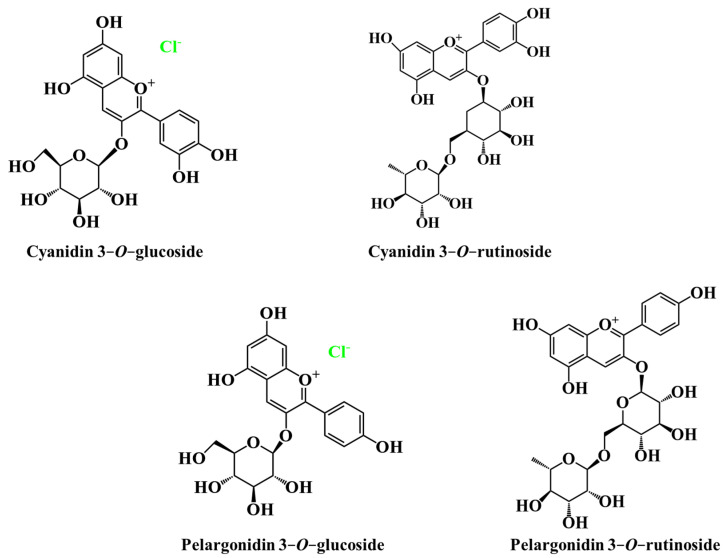
Principal anthocyanins present in *Rubus fruticosus*, *Rubus ulmifolius*, and *Morus nigra*.

Blackberry anthocyanins in *R. fruticosus* are mostly cyanidin derivatives (Figure 7). Cyanidin 3-*O*-glucoside is the most abundant anthocyanin found in blackberry at the ripened stage (92.3 to 335.6 mg per 100 g), followed by cyanidin 3-*O*-dioxalylglucoside (16.9–107.5 mg per 100 g) [39]. Other anthocyanins found in blackberry fruit include cyanidin 3-*O*-xyloside, cyanidin 3-*O*-dioxaloylglucoside, cyanidin 3-*O*-(6-malonyl)-glucoside, pelargonidin 3-*O*-glucoside, malvidin 3-*O*-glucoside, cyanidin 3-*O*-arabinoside, cyanidin 3-*O*-xyloside, cyanidin 3-*O*-dioxalylglucoside, and cyanidin-3-*O*-glucoside acylated with malonic acid [13,37,39,86]. Anthocyanins such as cyanidin 3-*O*-glucoside (92.3-335.6 mg per 100 g fw) and cyanidin 3-*O*-dioxalylglucoside (16.9-107.5 mg per 100 g fw) were also detected in *R. ulmifolius* [71].

On the other hand, mulberries present anthocyanin levels ranging from 184.3 to 227.0 mg per 100 g of fruit [87]. Among anthocyanins, cyanidin 3-*O*-glucoside, cyanidin 3-*O*-rutinoside, pelargonidin 3-*O*-glucoside, and pelargonidin 3-*O*-rutinoside are abundant in *M. nigra* [47].

According to research, the anthocyanin contents of blackberries vary depending on variety, environmental conditions, cultivation site, degree of ripeness, and processing. Fruit maturation is reported to influence the total amount of anthocyanin in blackberries. The antioxidant capacity peaks in some species at early stages of development. However, from a practical perspective, berries should be harvested when fully ripe because the maturity stage has a significant impact on their flavour and taste [11,33,39,41,49].

Regarding anthocyanins’ biological potential, it was reported that these phenolics have notable antioxidant abilities and capacity to induce enzyme activation, and hence inhibit possible DNA damage by carcinogens, reduce body inflammation, protect brain health, and enhance cognitive function [6].

According to existing data, the antioxidant potential of wild berries is higher than that of domesticated and genetically modified crops when comparing *R. fruticosus* and R. ulmifolius. In terms of anthocyanin content and antioxidant capacity, wild species are highly impressive. Anthocyanins are the main phenolic subclass found in *R. ulmifolius* fruits (23.8 mg per g extract), representing about 35% of the total phenolic compounds identified in them [13].

Evidence that anthocyanin values found in these fruits are higher when compared to other small fruits supports the enormous potential of blackberry and mulberry fruits as natural colour additives in the food, drug, nutraceuticals, and cosmetic industries, and their incorporation in pharmaceutics [83,84,86,88,89,90].

## 4. Health Benefits

Many studies have shown that the daily consumption of blackberries is an exceptionally essential source of health-promoting substances. Dietary improvements, particularly increased consumption of plant-based foods, may prevent more than 30% of all fatalities [91]. Blackberry fruit has been the subject of extensive research due to its high antioxidant content, which can normalize stress oxidative and inflammatory levels, as well as reduce cancer risk and cardiovascular complications, and has demonstrated biological activity against esophageal, colon, and oral cancers [24,92]. According to recent research, mulberries have positive biological properties that can help in the prevention of chronic diseases, such as cancer, neurotoxicity, obesity, diabetes, and memory loss [37,90,93].

The application in pharmaceutical sectors is critical for improving health naturally and without side effects. As far as we know, no negative effects of the administration of blackberries or mulberries have been observed, making it a feasible and potentially effective dietary strategy to improve disease prognosis [94].

### 4.1. Antidiabetic Properties

Diabetes mellitus is a chronic endocrine condition in which the pancreas either stops producing insulin or produces inadequate insulin. Diabetes affects about 425 million people globally and is defined by a rise in blood glucose concentration (>7 mmol/L) [95]. It has been associated with the development of various significant problems at cardiovascular, neurological, and renal levels, leading to increased morbidity and mortality [93]. The International Diabetes Federation anticipated that, by 2030, there will be 552 million diabetics globally [96].

To establish glycemic control, diabetics use insulin and other therapy drugs, such as metformin, sodium-glucose cotransporter-2 inhibitors, and glucagon-like peptide 1 [97]. Before the development of insulin, medicinal plants were used to treat this condition. Because of their low cost, availability, and lack of negative effects, the use of natural plants was and still is an alternative for many people. Various plant genera and phytochemical constituent types with anti-diabetic properties have been used in this context [31,45,97,98]. Therefore, it is not surprising that formulations using anti-diabetic plant extracts or phytocompounds have been derived. Additionally, nowadays, systems such as “Herbal-based anti-diabetic drug delivery systems” are largely used to provide herbal medicines to treat diabetes [98].

Certain regions of the world employ black mulberry leaves, fruits, and barks as anti-diabetic medications, believing in their efficacy in lowering blood glucose levels [31,99,100,101,102]. In accordance with this, *Morus nigra* has been shown to have a wide range of biological and pharmacological therapeutic benefits, including antidiabetic, anti-obesity, and anti-hyperlipidemic effects [103]. Hydroethanolic freeze-dried extracts of this fruit revealed potential for inhibiting pancreatic lipase, displaying a half maximal inhibitory concentration of 6.32 mg/mL [104].

Among both berries’ constituents, quercetin has been demonstrated to have considerable antioxidant and anti-inflammatory characteristics and the ability to interfere with a variety of antidiabetic activities, including insulin secretion and sensitization, glucose level improvement, and inhibition of intestinal glucose absorption. By activating adenosine monophosphate and preventing lipid peroxidation, this phenolic molecule promotes glucose transporter 4, the principal facilitative mediator of glucose uptake in skeletal muscles, adipose tissues, and other peripheral tissues. Given that, it is not surprising that quercetin can be used to stabilize blood glucose and body weight [105,106]. Furthermore, a single oral dosage of quercetin (400 mg) decreased *α*-glucosidase activity and reduced postprandial hyperglycemia in rats with type 2 diabetes [107].

Ferulic acid, another berry phenolic component, at 1000 mg per day for six weeks, showed the capacity to decrease total cholesterol, malonylaldehyde, TNF-*α*, and triglycerides by 8.1, 24.5, 13.1, and 12.1%, respectively, and increase HDL cholesterol by 4.3% [108]. These findings suggested that ferulic acid can also help diabetic patients with hyperlipidemia. Ferulic acid was found to be generally safe, with LD_50_ values of 2445 mg /kg in male rats and 2113 mg /kg in female rats [109].

Additionally, diabetic male Wistar rats received injections of black mulberry fruit extracts at 150 and 300 mg/kg body weight for 4 weeks. After this time, microalbuminuria, albumin, glucose, insulin, creatinine, and creatine levels in the serum were measured. The study discovered that diabetic animals considerably improved in all of the measures tested. The activity of catalase activity was also improved. Furthermore, the histological examination of their kidney tissues revealed a significant reduction in degenerative anomalies and glomerular sclerosis. TNF-*α*, vascular cell adhesion molecule-1, and fibronectin mRNA expression were all downregulated in treated rats [101]. Therefore, the downregulation of TNF-*α*, VCAM-1, and fibronectin levels in diabetic rats avoids, or retards, the development of diabetic nephropathy. Altogether, these data support the evidence that mulberry fruit extract may be a potential agent in the treatment of diabetic nephropathy [103].

### 4.2. Antimicrobial Properties

Plant-derived antimicrobial chemicals may limit the development of bacteria, fungi, viruses, and protozoa by different processes from those utilized by synthetic antimicrobials, and thus exhibit substantial therapeutic benefit in the treatment of resistant microbial strains. The antimicrobial activity of an agent is generally due its potential to chemically interfere with the manufacture or function of key components of bacteria and/or evade established antibacterial resistance mechanisms [45,110,111].

The majority of phytochemicals with therapeutic value found in fruits are secondary metabolites. Their antimicrobial activity varies depending on the structure, number, and position of substituent groups, the presence of glycosidic linkages, and the alkylation of hydroxyl groups [111,112]. As expected, blackberries’ antimicrobial properties differ among cultivars and ambient and soil factors. Furthermore, it is important to note that it is not possible to associate the antimicrobial activity with a specific compound due to the capacity of phenolic compounds to act synergistically [31,89,92,113,114,115,116,117,118].

Recent research has revealed that blackberries and mulberries have notable antimicrobial properties. The antimicrobial activity of different *R. fruticosus* extracts was investigated against *Escherichia coli*, *Staphylococcus aureus*, *Bacillus cereus*, *B. subtilis*, *B. mojavensis*, *Salmonella Hartford*, *Proteus vulgaris*, *Pseudomonas baetica*, *Micrococcus luteus*, and *Saccharomyces cerevisiae*. The inhibition zone diameter (mm) was measured, revealing that the ethanolic extracts are more competitive than the crude extracts, and show a notable antimicrobial potential against *Proteus vulgari* (20.53 mm). The lowest activity was observed against *S. Hartford* bacteria (9.54 mm). In this study, minimal inhibitory concentration (MIC) and minimum bactericidal concentration (MBC) were not calculated [113].

Additionally, hydroethanolic extracts of *R. ulmifolius* proved to have bacteriostatic effects against three Gram-negative bacteria (*E. coli*, *Morganella morganii*, and *P. mirabilis*), four Gram-positive bacteria (MRSA-methicillin-resistant *S. aureus*, MSSA-methicillin susceptible *S. aureus*, *Listeria monocytogenes*, and *Enterococcus faecalis*), and one fungus (*Candida albicans*). The results obtained in this work revealed activity in some tested strains, with MIC values ranging between 5 and >20 mg/mL. To inhibit the growth of *Klebsiella pneumoniae* and *Pseudomonas aeruginosa*, a concentration above 20 mg/mL was necessary. For the remaining Gram-negative strains, the most effective results were shown against *M. morganii* (MIC = 5 mg/mL) and *E. coli* (MIC = 5 mg/mL), followed by *P. mirabilis* (10 mg/mL) (Table 8) [13]. In another study, methanolic extracts of *R. ulmifolius* showed antimicrobial potential against two Gram-negative bacteria (*E. coli* and *Salmonella typhimurium*), three Gram-positive bacteria (*S. aureus*, *Enterococcus feacium*, *Streptococcus agalactiae*) and one fungus (*Candida albicans*). The most notable values were observed against *S. agalactiae* and *E. coli* bacteria (Table 8) [114].

The antimicrobial effects of *M. nigra* were also evaluated, especially in *S. aureus*, *P. aeruginosa*, and *E. coli*, where the ability of its extracts to inhibit the production of proinflammatory cytokines and interfere with iNOS and NF-κB pathways was observed [114]. Considering the higher content of anthocyanins in this species, these effects could be attributed to these compounds. In fact, anthocyanins have potent antiviral and antibacterial properties, being already known for their antimicrobial potential against *K. pneumonia*, *P. aeruginosa*, *S. aureus*, *E. coli*, H1N1, SARS-CoV-2, and rabies and herpes simplex virus [45,112,114].

Additionally, the antibacterial efficacy of mulberry total flavonols was assessed against three bacteria (*E. coli*, *P. aeruginosa*, and *S. aureus*), revealing interesting MBC results against *S. aureus* and *E. coli* (Table 8) [115]. Another investigation demonstrated the potential of *M. nigra* ethanolic extracts to be used in acne-treatment beauty care products given their capacity to inhibit *S. epidermis* and *P. acnes* growth, revealing MIC values of 2.5% for both bacteria, and MBC scores of 2.5% and 5% against *S. epidermidis* and *P. acnes*, respectively [116].

Black mulberry juice also has antibacterial properties, with its ability against three Gram-negative strains (*E. coli*, *P. aeruginosa*, and *S. typhimurium*) and five Gram-positive strains (*Bacillus spizizenii*, *B. subtilis*, *Corynebacterium diphtheriae*, *Enterococcus. faecalis*, and *S. aureus*) being previously reported. The maximum zone of inhibition was against *P. aeruginosa* (19.87 mm), followed by *Bacillus spizizenii* (19.68 mm) and *B. subtilis* (18.46 mm). The minimum zone of inhibition was obtained against *E. coli* (9.98 mm). Among the Gram-positive species, *Bacillus* species exhibited the highest zones of inhibition while, regarding Gram-negative bacteria, *P. aeruginosa* had higher inhibition than *S. typhimurium* and *E. coli* [117].

### 4.3. Antioxidant Activity

Reactive species are products of normal cellular metabolism and play key roles in signal transduction pathways, growth regulation, gene expression, and immune responses. In the human body, various mechanisms are necessary to maintain redox homeostasis [45,119]. These mechanisms include non-enzymatic and enzymatic antioxidant defenses created in the body (endogenous), as well as those given by the food (exogenous). However, the overproduction and accumulation of free radicals can lead to oxidative damage [6]. This biological condition may be caused by a lack of antioxidant defense mechanisms, excessive reactive species production, and excessive activation of their systems, increasing aging and the pathology of many chronic diseases, such as cancer, cardiovascular disease, inflammation, diabetes, and Parkinson’s and Alzheimer’s disease [45,120]. Therefore, it is essential to reduce their levels. Flavonoids, stilbenes, and tannins are examples of exogenous antioxidants. For example, scavenging and detoxifying radical oxygen species and preventing their production, influencing the cell cycle, avoiding tumor suppression, and modulating signal transduction, apoptosis events, and metabolism, are all biologically relevant mechanisms attributed to phenolic compounds [11,45,58,72,121,122,123,124]. Their antioxidant diversity and concentration are greatly dependent on the species and cultivars. Pre-harvest practices, environmental conditions, harvest ripeness, postharvest storage, and processing operations are also key drivers of phytochemical profiles [11,17,40].

Blackberries are considered one of the richest sources of natural antioxidants due to their high content of phenolic compounds, such as anthocyanins, ellagitannins, flavonols, and flavanols [13,41,44,49,51,62]. In fact, they present an extraordinary capacity to scavenge chemically generated radicals, thus preventing a wide range of human disorders and maintaining a healthy balance between free radicals and antioxidant systems. In particular, blackberries have notable antioxidant abilities against superoxide radicals (O_2_^●−^), hydrogen peroxide (H_2_O_2_), hydroxyl radicals (^●^OH), and singlet oxygen (^1^O_2_) [123].

The antioxidant capacity of blackberries was previously determined by in vitro assays, by the lipid peroxidation inhibition assays (TBARS), oxidative reactive oxygen and nitrogen species (ROS/RNS), hemolysis inhibition assay, the ORAC method, 2,2′-azinobis (3-ethylbenzothiazoline-6-sulphonic acid (ABTS^•+^), the ferric-reducing/antioxidant power (FRAP) method, 2,2-diphenylpicrylhydrazyl (DPPH^•^), and Trolox equivalent antioxidant capacity (TEAC) assay [23,37,39,49,52,56,71,89,89,125,126,127,128,129,130,131,132].

In the TBARS experiment, *R. fruticosus* extract revealed a high antioxidant activity, displaying an IC_50_ value of 100 ug/mL, which is substantially lower than that obtained with the positive control, Trolox (139 ug/mL) [49]. Additionally, using FRAP assay, ABTS^•+^, and DPPH^•^, the obtained results were between 4.45–14.16 for FRAP, 2.28–8.89 for ABTS^•+^, and 2.63–9.35 mmol Trolox equivalents per⋅100 g fw for DPPH^•^ [71].

Furthermore, methanolic extracts of *M. nigra* at 76 µg showed the capacity to inhibit lipid oxidation by 28.7%, while ethanolic extracts exhibited lower inhibitory capacity (23.7–47.6%) [125]. The antioxidant abilities of its aqueous extracts were also evaluated, revealing lower abilities than the methanolic ones; at 100 µg/mL, the values obtained were 1.1% and 7.1% for aqueous and methanolic extracts, respectively, whereas at 300 µg/mL, the corresponding values were 7.1% and 21.6%, respectively [125].

However, when comparing wild blackberries (*R. ulmifolius*) with the cultivated ones (*R. fruticosus*), substantial differences were found, with the latter having higher antioxidant content [126].

To summarize, mulberries have lately gained a large amount of interest as prospective sources of functional foods due to a variety of biological benefits [103,133]. The obtained findings on the antioxidant activity of mulberry fruits support their incorporation in biological applications [100,103,125,130].

### 4.4. Anti-Inflammatory Properties

Inflammation is the immune system’s reaction to potentially damaging stimuli such as infection or injury. In the presence of stressors, immune cells release inflammatory substances, such as inflammatory cytokines, including TNF-*α* and interleukins (IL)-6 and 10, leading to increased nitric oxide (NO) levels and prostaglandins via the catalysis of cyclooxygenase-2 (Cox2) and NF-κB pathways [45,134,135]. Blackberry freeze-dried powders are capable of reducing mRNA expression of NF-kB and COX-2 in the liver [136].

A healthy lifestyle that includes physical activity, stopping smoking, and moderate alcohol intake, associated with a diet rich in fruits, vegetables, and whole grains, decreases the risk of developing chronic diseases. As expected, phenolic compounds, carotenoids, vitamins, and dietary fiber contribute to the anti-inflammatory and antioxidant effects of fruits and vegetables [45,48,137,138,139]. In particular, high quantities of dietary anthocyanins may be viewed as a feasible nutraceutical in the context of inflammatory disease. Among these, cyanidin 3-*O*-glucoside can reduce cytokine-induced inflammation in intestinal cells by inhibiting the production of NO, PGE2, and IL-8, and the expression of iNOS and COX-2 [112,138,139,140].

Focusing on blackberries and mulberries, anthocyanin-enriched fractions from fermented blueberry and blackberry beverages inhibited dipeptidyl peptidase-IV activity in LPS-stimulated murine macrophages. Computational docking demonstrated that this effect could be mainly attributed to delphinidin 3-*O*-arabinoside, which effectively inactivates dipeptidyl peptidase-IV by binding with a low interaction energy (−3228 kcal/mol). Additionally, anthocyanins and proanthocyanidins (100 µM cyanidin 3-*O*-glucoside and epicatechin equivalents, respectively) extracted from them reduced LPS-induced inflammatory response in mouse macrophages by stopping the NF-κB pathway [140]. Another study that used RAW 264.7 macrophages stimulated with LPS demonstrated that blackberry anthocyanin extract (0–20 µg/mL)-treated macrophages presented lower levels of IL-1 and TNF-*α* [141]. Once again, this reduction is mainly associated with the ability of anthocyanins to interfere with NF-κB signaling [140], particularly of cyanidin 3-*O*-glucoside, which previously showed potential to decrease pro-inflammatory mediators NO, PGE2, COX-2, and IL-8 generated by cytokine-stimulated HT-29 cells [139]. In accordance with this, *R. fruticosus* also showed capacity to inhibit the secretion of pro-inflammatory IL-8 cytokines in two cellular models (HT-29 and T-84 cells) in a dose dependent-manner in both cell lines [92].

Ellagitannins are another significant polyphenol that has displayed anti-inflammatory properties. Previous research [142] examined their anti-inflammatory efficacy of TNF-*α*, IL-1B, IL-8, and NF-κB on the AGS gastric cell line. Ellagitannins extracted from *R. fruticosus* suppressed TNF-*α*, showing an IC_50_ value of 0.67–1.73 mg/mL. At 2 mg/mL, ellagitannins inhibited TNF-*α* and NF-κB nuclear translocation by 57% and 67%, respectively. At lower doses, ellagitannins reduced IL-8 secretion, revealing an IC_50_ ranging between 0.7 and 4 mg/mL. Moreover, in a rat model of ethanol-induced stomach lesions, the protective effect of ellagitannins was also tested. Ellagitannins (20 mg/kg/day) were administered orally to rats for ten days, and ethanol was administered one hour before sacrifice. The mucosa of the stomach was separated and utilized to measure IL-8 release, NF-κB nuclear translocation, TEAC, and superoxide dismutase and catalase activities. This investigation demonstrates that the treatment with these compounds can decrease NF-κB nuclear translocation and suppress IL-8 production. The present work demonstrated that ellagitannins derived from *Rubus* berries definitively protect against the formation of gastric ulcers in rat animal models. In particular, ellagitannins can block the NF-κB cascade either directly on the cell response to pro-inflammatory cytokines or act as antioxidant agents by inhibiting reactive species generated in several inflammatory conditions [143].

### 4.5. Neuroprotection

The human brain is responsible for a wide range of cognitive, motor and behavioral functions that require significant amounts of energy. Neurons are responsible for transmitting information to and from the brain. Neurodegenerative illnesses are distinguished by progressive brain cell death and neuronal loss, which impair motor or cognitive function. Alzheimer’s disease, Parkinson’s disease, Huntington’s disease, amyotrophic lateral sclerosis, and spinocerebellar ataxia are examples of common neurodegenerative disorders. These disorders are a major public health concern, particularly among the elderly [120]. These disorders develop because the brain is more sensitive to oxidative stress than other organs due to the poor activity of antioxidant defense mechanisms [144].

Many epidemiological studies are being conducted to study the potential of phenolics to be used to promote neuronal health and prevent neural cells from being damaged through their antioxidant and anti-inflammatory properties, and thus delay Parkinson’s and Alzheimer’s diseases, ischemic diseases, and aging effects [137,145].

The inclusion of blackberries in the diet has been shown to reduce brain degeneration [9,93,146,147,148]. The neuroprotective capacity of this fruit mainly comes from its antioxidant capacity, promoted by the presence of phenolic compounds, such as anthocyanins, caffeic acid, and quercetin [45,50,137,148,149,150]. Indeed, these compounds can penetrate the hematoencephalic membrane and have neuroprotective effects on various cerebral structures in the brain, including the hippocampus and cortex [122]. Flavonoids may also have important impacts on mammalian cognitive function, perhaps halting the aging-related declines in memory and learning. These benefits are mostly sought for preventing brain damage, such as neurodegenerative diseases, and improving memory, learning, and cognitive functions [148]. Blackberries from the north of Portugal can lower intracellular reactive species levels, alter glutathione levels, and inhibit the emergence of caspases during treatment, hence reducing oxidative stress and preventing neurodegeneration [147]. Mulberry fruit extracts and cyanidin 3-*O*-glucoside have shown the ability to inhibit reactive species production and, consequently, neuronal injury [151,152].

Neuroblastoma cells exposed to H_2_O_2_ and treated with raw, digested, and dialyzed blackberry extracts at physiological concentrations revealed lower age-related neurodegeneration [9]. In addition, animal research found that an intermediate dosage of blackberry juice (5.83 mg/kg anthocyanins, 27.10 mg/kg polyphenols) enhanced mechanisms of behavioral coping with diazepam l. The forced swim test supported these findings by demonstrating that blackberry juice, at moderate and high doses, improves the acute stress response [153]. These findings suggest that blackberry juice may have a therapeutic value in alleviating anxiety caused by stressful experiences.

*M. nigra* revealed a notable protective effect against Alzheimer’s disease, specifically by inhibiting amyloid-*β*-induced paralysis symptoms and suppressing over-sensitivity to exogenous serotonin by about 55.65% in transgenic Alzheimer’s disease *Caenorhabditis elegans* models, which were treated with up to 1.00 mg/mL. These effects are due to the capacity of this fruit to activate the DAF-16/SOD-3/GST-4 pathway, improve antioxidant capacity, delay aging, and alleviate the symptoms caused by the amyloid-*β* protein [154]. These findings suggest that functional foods, such as mulberry, can be used to lower the risk of Alzheimer’s disease.

### 4.6. Anticancer Activity

Cancers are characterized by abnormal cell growth capable of invading other regions of the body, resulting in metastasis. A tumor is a complex multistage process that begins with the genesis of a cancer cell caused by DNA damage, followed by accumulation of mutations, progression to cell proliferation and tumor expansion, and, finally, progression to malignancy and metastasis. While new cancer incidence is expected to rise by 70% by 2034, approximately 35% of cancer deaths are attributed to behavioral and dietary risks, such as high body mass index, low fruit and vegetable intake, and lack of physical activity [155].

According to epidemiological and clinical research, a diet consisting of 400–800 g of various vegetables and fruits per day can prevent 20% or more of all cancer cases [2,48,137].

Phenolic berry content has shown the capacity to reduce inflammation, inhibit angiogenesis, protect against DNA damage, and influence apoptosis or proliferation rates in malignant cells. Indeed, they demonstrate the ability to interfere in all phases of cancer development, including initiation, promotion, progression, invasion, and metastasis [45,134,150,156]. Berry extracts also inhibited cancer-induced AP-1 and NF-κB, as well as decreasing the expression of the two proteins involved in tumor promotion and progression, i.e., vascular endothelial growth factor and COX2 [136,157]. These effects are intimately linked to the capacity of phenolics to alter the genomic stability at many phases in the cancer genesis process [137]. For example, anthocyanins have been shown to activate phase II enzymes, which may inactivate carcinogens triggered by phase I enzymes, and hence prevent DNA damage caused by the carcinogens [82,112,124,158].

Dietary bioactive compounds can also decrease telomerase activity by modifying histones or by inhibiting DNA methyltransferases. Telomerase activity has been detected in more than 80% of human malignancies, making the enzyme a promising target for anticancer treatment. According to research, the antiproliferative impact of blackberry fruits is mediated by their anti-telomerase activity [159]. Additionally, there have been no negative effects associated with the administration of blackberries, indicating that this fruit has the potential to be effective for a dietary plan to reduce cancer risk and assist cancer patients with illness prognosis [157].

Blackberries previously demonstrated significant chemo-preventative and antioxidant activities by inhibiting the growth, proliferation, and migration of the human A549 lung carcinoma cell line, and strong inhibitory effects on the cell growth of highly metastatic breast cancer HS578T cells, by inducing significant alterations in cell cycle regulators, causing G2/M arrests [160]. Blackberries and mulberries contain anthocyanin cyanidin-3-*O*-glucoside, which has promising qualities for usage in nutraceuticals, and has shown potential to limit cell proliferation, arresting the cell cycle in the G2/M phase, and inducing apoptosis in vitro [112,138,161]. In fact, in a recent investigation, rats were administered orally with purified cyanidin 3-*O*-glucoside (800 µmol/kg of body weight). After 30 min–2.0 h of delivery, this was detected in plasma, with a C_max_ value of 0.8 µM. This evidence represents added value regarding the incorporation of this anthocyanin in dietary supplements, aiding in the anticancer therapy of breast cancer [161].

*Morus nigra* extracts have also been the subject of much research. A three-month enriched diet applied in MUC2^−/−^ mice, with a model of spontaneous chronic intestinal inflammation and induced-intestinal tumors at three months, at 5% or 10%, resulted in a reduction in tumorigenesis and intestinal inflammation. Basically, mice aged 6 to 8 weeks that were supplemented with 5% or 10% *M. nigra* extracts for 10 days and there were observed improvements in their signs and symptoms caused by dextran sulfate sodium-induced acute colitis, preventing weight loss and bloody stools, and promoting positive changes in the histology of the colorectal lining [162].

### 4.7. Cardiovascular Protection

Cardiovascular disorders affect the heart and blood vessels and are the major cause of death worldwide. People who have high blood pressure and cholesterol, as well as smokers, those who are sedentary or obese, and people who have a diet rich in salt, sugar, and fatty acids, are more susceptible to cardiovascular problems [163].

The current nutritional guidelines for the prevention of cardiovascular diseases include a Mediterranean-style diet rich in fruits, vegetables, and whole grains, as well as non-tropical vegetable oils, in order to reduce total cholesterol, oxidative stress, and inflammation [2,48,50,59,137,164].

Blackberry phenolic compounds have demonstrated the capacity to diminish LDL oxidation, quench free radicals by hydrogen molecule donation, and interfere with liposome oxidation systems [165,166]. In particular, anthocyanins from *M. nigra* showed the capacity to protect human primary endothelial cells by decreasing the production of the cytokine-induced chemokine monocyte chemotactic protein 1, a protein directly linked to atherogenesis, and which is mainly responsible for attracting macrophages to sites of infection or inflammation [167]. Moreover, although not directly shown in blackberry flavonoids, several flavonoids also revealed the capacity to protect platelet function, which is crucial in the pathogenesis of these diseases. In fact, flavonoids can minimize platelet aggregation, reduce platelet generation of superoxide anions, and increase platelet NO production [168].

In epidemiological studies, diets high in plant-derived phenolic compounds have been shown to reduce the incidence of coronary heart disease. The chronic antioxidant and hypolipidemic characteristics of these compounds play critical roles in the prevention of lipoprotein oxidation and the formation of atherosclerotic lesions [2,122,166,169].

## 5. Conclusions

The health benefits of fruits vary based on their composition, growth, and environmental circumstances. Mulberries and blackberries are little red/purple fruits that have high levels of natural health-promoting chemicals. These fruits are rich in phytochemicals, such as anthocyanins, ellagitannins, flavanol glycosides, and phenolic acids, as well as dietary fiber. All of these are beneficial to human health and fitness. Several studies have demonstrated that the phytochemical contents of *R. fruticosus*, *R. ulmifolius*, and *M. nigra* can act as antioxidant, anti-inflammatory, neuroprotector, and antitumoral agents, and offer cardiovascular protection. However, further studies are needed to completely understand the mechanism of action of the blackberry and mulberry metabolites that trigger the biological activities outlined in this review. Furthermore, more in vitro and in vivo studies are also required to assess the impact of daily consumption of these small fruits and to determine their optimal doses to maximize human health benefits. New understanding must be created in order to build novel medications for future pharmaceutical and nutraceutical uses.

## Figures and Tables

**Table 1:** Basic chemical composition, macronutrients, and mineral content of blackberry and mulberry (per 100 g of fresh weight) [41,47,48].

Nutrient (Unit)	Basic Chemical Composition	
	Raw Blackberry	Raw Black Mulberry
**Water (g/100 g)**	88.2	87.7
**Energy (kcal/100 g)**	43–125.25	43
**Macronutrients**		
**Protein (g/100 g)**	1.39–2.4	1.44
**Total lipid (fat)**	0.49–1.22	0.39
**Fatty acids, total monounsaturated (g/100 g)**	0.047	0.041
**Fatty acids, total polyunsaturated (g/100 g)**	0.28	0.207
**Ash (g/100 g)**	0.37–0.58	0.69
**Carbohydrate, by difference (g/100 g)**	9.61–26.2	9.8
**Dietary fiber (g/100 g)**	5.3	1.7
**Total sugars (g/100 g)**	4.78–16.3	10.14–21.32
**Sucrose (g/100 g)**	0.07–0.34	1.08–2.14
**Glucose (g/100 g)**	2.31–8.1	7.18–10.33
**Fructose (g/100 g)**	2.4–7.8	1.88–8.85
**Maltose (g/100 g)**	0.07	-
**Galactose (g/100 g)**	0.03	-
**Micronutrients**		
**Minerals**		
**Calcium, Ca (mg/100 g)**	12.5–29	39–502
**Iron, Fe (mg/100 g)**	0.62–3.4	1.85–77.6
**Magnesium, Mg (mg/100 g)**	20	18–386
**Phosphorus, P (mg/100 g)**	22	38–2520
**Potassium, K (mg/100 g)**	11.9–162	194–2234
**Sodium, Na (mg/100 g)**	1	5.9–302
**Zinc, Zn (mg/100 g)**	0.53	0.10–62
**Cooper, Cu (mg/100 g)**	0.165	0.06–0.10
**Manganese, Mn (mg/100 g)**	0.646	0.40–19
**Selenium, Se (µg/100 g)**	0.4	0.008–0.6

**Table 2:** Organic acids identified in *Rubus ulmifolius* and *Rubus fruticosus* blackberries, and *Morus nigra* mulberry [13,36,51,52].

Organic Acid	*R. ulmifolius*	*R. fruticosus*	*M. nigra*
**Citric acid**	-	125.54 mg per 100 g dw	1084–7020 mg per 100 g fw
**Oxalic acid**	71 mg per 100 g fw	59.51 mg per 100 g dw	450–1250 mg per 100 g fw
**Quinic acid**	119 mg per 100 g fw	-	-
**Malic acid**	29 mg per 100 g fw	5706.37 mg per 100 g dw	1323–13,650 mg per 100 g fw
**Succinic acid**	-	-	342 mg per 100 g fw
**Shikimic acid**	11.33 mg per 100 g fw	-	1.36 mg per 100 g fw
**Tartaric acid**	-	-	220–860 mg per 100 g fw
**Ascorbic acid**	6.66 mg per 100 g fw	6.00 mg per 100 g dw	12.81–15.37 mg per 100 g fw
**Fumaric acid**	tr	230.25 mg per 100 g dw	-
**Total**	238 mg per 100 g fw	6127.67 mg per 100 g dw	2951 mg per 100 g fw

tr: traces; -: no data; fw: fresh weight; dw: dry weight.

**Table 3:** Vitamin content of raw blackberry and mulberry fruits [30,33,41,43,47,48,56,57].

Vitamins	Raw Blackberry	Raw Black Mulberry
**Vitamin C (mg/100 g)**	21.0	19.3–36.4
**Thiamin (mg/100 g)**	0.02	0.029
**Riboflavin (mg/100 g)**	0.026	0.04–0.10
**Niacin (mg/100 g)**	0.646	0.62–1.60
**Vitamin B-6 (mg/100 g)**	0.03	0.05
**Folate total (µg /100 g)**	25.0	6.0
**Folate, DFE (µg /100 g)**	25.0	6.0
**Folate, food (µg /100 g)**	25.0	6.0
**Choline, total (mg/100 g)**	8.5	12.3
**Vitamin K (phylloquinone) (µg /100 g)**	19.8	7.8

**Table 4:** Tocopherols present in in *Rubus ulmifolius* and *Rubus fruticosus* blackberries, and *Morus nigra* mulberry [13,49,58].

Tocopherols	*R. ulmifolius* (mg per 100 g fw)	*R. fruticosus* (mg per g Extract)	*M. nigra* (mg per g Extract)
***α*** **-tocopherol**	1.15–3.38	6.1	43
***β*** **-tocopherol**	0.02–0.24	nd	1.27
***γ*** **-tocopherol**	1.34–3.73	nd	12.5
***δ*** **-tocopherol**	0.9–3.69	nd	5.5
**Total**	5.1–13.48	6.1	62

nd: not detected; fw: fresh weight.

**Table 5:** Carotenoids present in *Rubus fruticosus* blackberry and *Morus nigra* mulberry [47].

Carotenoids	*R. fruticosus*	*M. nigra*
**Carotene, beta (µg per 100 g)**	128.0	9.0
**Carotene, alfa (µg per 100 g)**	0.0	12.0
**Vitamin A, RAE (µg per 100 g)**	11.0	1.0
**Vitamin A, IU (µg per 100 g)**	214.0	25.0
**Lutein + zeaxanthin (µg per 100 g)**	118.0	136.0

**Table 6:** Volatile compounds identified in *R. fruticosus*, *R. ulmifolius*, and *M. nigra* fruits [62,64,65,66].

Volatile Compounds	Fruit Species	Volatile Compounds	Fruit Species
Esters			
Methoxyphenyl oxime	*R. ulmifolius*	Methyl salicylate	*R. ulmifolius*
Ethyl octanoate	*R. ulmifolius*	Methyl acetate	*M. nigra*
Etyl acetate	*M. nigra*	Etyl propanoate	*M. nigra*
Etyl 2-metylbutanoate	*M. nigra*	Propyl acetate	*M. nigra*
Ethyl 3-metylbutanoate	*M. nigra*	Etyl butanoate	*M. nigra*
Isopentyl acetate	*M. nigra*	Etyl pentanoate	*M. nigra*
Ethyl 2-hydroxyhexanoate	*M. nigra*	Ethyl lactate	*M. nigra*
Isoamyl lactate	*M. nigra*	Ethyl octanoate	*M. nigra*
Ethyl decanoate	*M. nigra*	Ethyl 9-decenoate	*M. nigra*
Diethyl succinate	*M. nigra*	Benzyl acetate	*M. nigra*
2-Phenylethyl acetate	*M. nigra*	Methyl salicytate	*M. nigra*
Ethyl dodecanoate	*M. nigra*	Diethyl pentanedioate	*M. nigra*
Ethyl-3phenylpropanoate	*M. nigra*	Ethyl phenoylethanoate	*M. nigra*
Ethyl tetradecanoate	*M. nigra*	Ethyl hexadecanoate	*M. nigra*
Metyl-hexanoate	*R. futicosus*	Ethyl-hexanoate	*R. fruticosus* *M nigra*
Ethyl benzoate	*R. futicosus*	Methyl salicylate	*R. futicosus*
**Terpenes**			
D-limonene	*R. ulmifolius*	b-Linalool	*R. ulmifolius*
L-*α*-terpineol	*R. ulmifolius*	b-Myricene	*R. ulmifolius*
**Terpenoids**			
*α*-Thujene	*R. futicosus*	*β*-Myrcene	*R. futicosus*
*α*-Pinene	*R. futicosus*	*α*-Phellandrene	*R. futicosus*
1-Octanol	*R. futicosus* *M. nigra*	Terpinolene	*R. futicosus*
Camphene	*R. futicosus*	Limonene	*R. futicosus*
o-Cimene	*R. futicosus*	*α*-Terpinene	*R. futicosus*
Linalool	*R. futicosus*	Linalool oxide	*R. futicosus*
***trans*** Limonene oxide	*R. futicosus*	Isoborneol	*R. futicosus*
Isopinocarveol	*R. futicosus*	Terpinen-4-ol	*R. futicosus*
(-)-Carvone	*R. futicosus*	*p*-Cymen-8-ol	*R. futicosus*
Geraniol	*R. futicosus*	*α*-Copaene	*R. futicosus*
Vitispirane	*R. futicosus*	*α*-Terpineol	*R. futicosus*
Theaspirane	*R. futicosus*		
**Aldehydes**			
Pentanal	*R. ulmifolius*	Hexanal	*R. futicosus* *R. ulmifolius*
E-2-Pentenal	*R. ulmifoliu*	Nonanal	*R. futicosus* *R. ulmifolius*
E-2-Hexenal	*R. ulmifolius*	Z-2-Heptenal	*R. ulmifolius*
2-Hexenal	*R. futicosus*	Octanal	*R. futicosu*
Heptanal	*R. futicosus*	Decanal	*R. futicosus*
Nonenal	*R. futicosus*	p-Mentenal	*R. futicosus*
Acetaldehyde	*M. nigra*	Benzaldehyde	*R. futicosus* *M. nigra*
**Alcohols**			
2-Ethyl-1-pentanol	*R. ulmifolius*	Phenylthyl alcohol	*M. nigra*
1-Penten-3-ol	*R. ulmifolius*	1-Octen-3-ol	*R. ulmifolius*
Isoamyl alcohol	*R. ulmifolius*	Sulcatol	*R. ulmifolius*
2-Heptanol	*R. ulmifolius* *R. futicosus* *M. nigra*	(s)-3-Ethyl-4- methylpentanol	*R. ulmifolius*
Z-2-Penten-ol	*R. ulmifolius*	Z-5-Octen-1-ol	*R. ulmifolius*
1-Hexanol	*R. ulmifolius* *M. nigra*	Benzyl alcohol	*M. nigra* *R. ulmifolius*
1-Heptanol	*R. futicosus* *R. ulmifolius*	E-2-Hexen-1-ol	*R. ulmifolius*
Z-3-Hexen-1-ol	*R. ulmifolius*	2-Tetradecanol	*M. nigra*
2-Butanol	*M. nigra*	2-Pentadecanol	*M. nigra*
1-Propanol	*M. nigra*	2-Nonanol	*M. nigra*
3-Methyl-2-butanol	*M. nigra*	1-Octanol	*M. nigra* *R. fruticosus*
2-Metyl-1-butanol	*M. nigra*	4-Methyl-1-pentanol	*M. nigra*
3-Methyl-1-butanol	*M. nigra*	3-Methyl-1-pentanol	*M. nigra*
3-Methyl-3-buten-1-ol	*M. nigra*	Terpene-4-ol	*M. nigra*
1,3-Butanediol	*M. nigra*	2-Decanol	*M. nigra*
2-Undecanol	*M. nigra*	Ethanol	*M. nigra*
2-Methyl-1-propanol	*M. nigra*	2,3-Butanediol	*M. nigra*
2-Butyl-1-octanol	*M. nigra*	3-Ethyl-4-methyl-pentanol	*M. nigra*
**Ketones**			
Methyl ethyl ketone	*R. futicosus*	Damascenone	*R. futicosus*
2-Heptanone	*R. futicosus*	Verbenone	*R. futicosus*
3-Hydroxy-2-butanone	*M. nigra*		
**Hydrocarbons**			
Pentadecane	*M. nigra*	Dodecane	*M. nigra*
Nonadecane	*M. nigra*	Tridecane	*M. nigra*
Heptane	*R. futicosus*	Tetradecane	*M. nigra*
Toluene	*R. futicosus*		
**Acids**			
Hexanoic acid	*M. nigra*	Acetic acid	*M. nigra*
Octanoic acid	*M. nigra*	Butanoic acid	*M. nigra*
Isovaleric acid	*M. nigra*		
**Carbonyls**			
1-Penten-3-one	*R. ulmifolius*	2-Heptanone	*R. ulmifolius*
Sulcatone	*R. ulmifolius*	2-Methyl butanoic acid	*R. ulmifolius*
**Phenols**			
2,4-Di-tert-butylphenol	*M. nigra*	2-Methoxyphenol	*M. nigra*
4-Methyl-2-methoxyphenol	*M. nigra*		
**Acids**			
Hexanoic acid	*M. nigra*	Acetic acid	*M. nigra*
Octanoic acid	*M. nigra*	Butanoic acid	*M. nigra*
Isovaleric acid	*M. nigra*		

**Table 7:** Phenolic compounds reported in *Rubus fruticosus*, *Rubus ulmifolius*, and *Morus nigra*.

Phenolic Compounds	*R. fruticosus*	*R. ulmifolius*	*M. nigra*	References
Phenolic Acids				
**Hydroxybenzoic acids**				
*p*-Hydroxybenzoic acid	1.44 mg per 100 g fw	-	0.053–0.47 mg per 100 g dw	[36,65]
Gallic Acid	145.85 mg per 100 g fw	268.72 mg per 100 g fw	21.83–40.90 mg per 100 g fw	[44,65,68]
Syringic acid	-	40.84 µg per 100 g dw	-	[69]
Vanillic acid	14.72 mg per 100 g	-	0.014–0.10 mg per 100 g dw	[37,68]
Salicylic acid	-	296.62 µg per 100 g dw	0.007–0.12 mg per 100 g dw	[37,69]
Ellagic acid	30.01–33.81 mg per 100 g fw	-	1.36–6.32 mg per 100 g fw	[44,70]
**Hydroxycinnamic acids**				
Caffeic acid	-	75.52 µg per100 g dw	6.14–21.93 mg per 100 g fw	[44,69]
Ferulic acid	2.99–22.09 mg per 100 g fw	388.59 µg per 100 g dw	0.009–00.056 mg per 100 g dw	[37,69,70]
Chlorogenic acid	-	-	43.76–97.59 mg per 100 g fw	[44]
*p*-Coumaric acid	0.40–2.08 mg per 100 g fw	39.65 µg per 100 g dw	-	[69,70]
Sinapic acid	-	228.69 µg per 100 g dw	0.013–0.11 mg per 100 g dw	[37,69]
**Flavonoids**				
**Flavonols**				
Quercetin	20.62 mg per 100 g fw	5509.61 µg per 100 g dw	2.33–11.25 mg per 100 g fw	[44,65,69]
Rutin	4.16–6.45 mg per 100 g	-	32.06–133.60 mg per 100 g fw	[44,68]
Quercetin 3-*O*-galactoside	5.44 mg per 100 g fw	-	-	[71]
Quercetin 3-*O*-glucoside	18.18 mg per 100 g fw	36.46 mg per 100 g	-	[68,71]
Kaempferol	0.63 mg per 100 g	399.48 µg per 100 g dw	0.009–0.17 mg per 100 g dw	[37,68,69]
**Flavan-3-ols**				
(+)-Catechin	265.75–312.86 mg per 100 g fw	156.61 µg per 100 g dw	2.28–10.54 mg per 100 g fw	[44,69,70]
(+)-Epicatechin	-	250.82 µg per 100 g dw	0.004–0.054 mg per 100 g dw	[37,69]
(-)-Epicatechin	94.29 mg per 100 g fw	-	-	[65]
**Flavone**				
Myricetin	9.99 mg per 100 g fw	-	-	[70]
Luteolin	-	5.97 µg per 100 g dw	0.098–2.26 mg per 100 g dw	[37,69]
**Flavanone**				
Naringenin	-	28.34 µg per 100 g dw	-	[69]
**Anthocyanins**				
Cyanidin 3-*O*-glucoside	19.49–86.73 mg per 100 g fw	92.3-335.6 mg per 100 g	6.01 mg per g extract	[39,49,71]
Cyanidin *O*-hexoside	-	3.76 mg per g extract	-	[49]
Cyanidin 3,5-diglucoside	55,447.28 µg per 100 g	-	0.51–7.28 mg per 100 g dw	[37,72]
Cyanidin 3-*O*-rutinoside	330,616.73 µg per 100 g	-	1.00–9.21 mg per 100 g dw	[37,72]
Cyanidin *O*-rhamnoside-*O*-hexoside	-	-	2.43 mg per g extract	[49]
Cyanidin *O*-pentoside	-	1.27 mg per g extract	-	[49]
Cyanidin 3-*O*-xyloside	2.62 mg per g extract	12.1–47.1 mg per 100 g	-	[13,39]
Cyanidin 3-*O*-malonylglucoside	-	5.7–20.9 mg per 100 g	-	[39]
Cyanidin 3-*O*-dioxalylglucoside	1.20–2.04 mg per g extract	16.90–107.50 mg per 100 g	-	[39,71]
Delphinidin 3-*O*-glucoside	-	-	0.24–7.42 mg per 100 g dw	[37]
Pelargonidin 3-*O*-glucoside	102,936.30 µg per 100 g	-	0.012–0.068 mg per 100 g dw	[37,72]
Pelargonidin 3-*O*-rutinoside	4.23 mg per 100 g fw	-	-	[71]

-: no data; fw: fresh weight; dw: dry weight.

**Table 8:** Antimicrobial effect of *Morus nigra* juice, *Rubus fruticosus* (crude and ethanolic extracts), and *Rubus ulmifolius* (methanolic and hydroethanolic extracts) [13,113,114,115,116,117].

Antimicrobial Activity								
Microorganisms	**M. nigra* juice* (100 µL)	*R. fruticosus*		*R. ulmifolius*				
		Crude Extract	Ethanolic Extract	Methanolic Extract (15 µL)			Hydroethanolic Extract	
	Mean Zone of Inhibition (mm)				MIC	MBC	MIC	MBC
Gram-negative bacteria								
*Escherichia coli*	9.98	9.37	16.70	28	4.03	8.92	5	>20
*Klebsiella pneumoniae*	-	-	-	-	-	-	>20	>20
*Morganella morganii*	-	-	-	-	-	-	5	>20
*Porteus mirabilis*	-	-	-	-	-	-	10	>20
*Proteus vulgaris*	-	12.75	20.53	-	-	-	-	-
*Pseudomonas aeruginosa*	19.87	-	-	-	-	-	>20	>20
*Pseudomonas baetica*	-	9.76	14.30	-	-	-	-	-
*Salmonella typhimurium*	11.73	-	-	22.5	4.13	8.24	-	-
*Salmonella Hartford*	-	14.49	9.54	-	-	-	-	-
**Gram-positive bacteria**								
*Enterococcus faecium*	-	-	-	16	4.76	8.70	-	-
*Enterococcus faecalis*	16.03	-	-	-	-	-	5	>20
*Listeria monocytogenes*	-	-	-	-	-	-	5	>20
*Bacillus spizizenii*	19.68	-	-	-	-	-	-	-
*Bacillus cereus*	-	11.20	14.00	-	-	-	-	-
*Bacillus subtilus*	18.46	8.10	14.04	-	-	-	-	-
*Bacillus mojavensis*	-	9.79	15.43	-	-	-	-	-
*Corynebacterium diphtheriae*	15.57	-	-	-	-	-	-	-
*Micrococcus luteus*	-	10.64	15.00	-	-	-	-	-
*Saccharomyces cerevisiae*	-	-	11.52	-	-	-	-	-
*Staphylococus aureus*	17.37	7.28	15.64	39	3.22	7.17	-	-
*Streptococcus agalactiae*	-	-	-	50	2.29	4.38	-	-
MRSA	-	-	-	-	-	-	10	>20
MSSA	-	-	-	-	-	-	-	>20
**Fungi**								
*Candida albicans*	-	-	-	39	-	-	-	-

-: no data; MIC: minimal inhibitory concentration; MBC: minimum bactericidal concentration.

## Data Availability

Not applicable.
